# 17α-ethynylestradiol prevents the natural male-to-female sex change in gilthead seabream (*Sparus aurata* L.)

**DOI:** 10.1038/s41598-020-76902-9

**Published:** 2020-11-18

**Authors:** M. Pilar García Hernández, Isabel Cabas, M. Carmen Rodenas, Marta Arizcun, Elena Chaves-Pozo, Deborah M. Power, Alfonsa García Ayala

**Affiliations:** 1 Department of Cell Biology and Histology, Faculty of Biology, University of Murcia, Campus de Espinardo, 30100 Murcia, Spain; 2 Oceanographic Center of Murcia, Spanish Institute of Oceanography (IEO), Carretera de la Azohía s/n, Puerto de Mazarrón, 30860 Murcia, Spain; 3 Centro de Ciências Do Mar, Universidade Do Algarve, Campus de Gambelas, 8005-139 Faro, Portugal

**Keywords:** Endocrine reproductive disorders, Marine biology, Animal physiology, Ichthyology, Endocrinology, Risk factors

## Abstract

Exposure to 17α-ethynylestradiol (EE_2_, 5 μg/g food) impairs some reproductive events in the protandrous gilthead seabream and a short recovery period does not allow full recovery. In this study, spermiating seabream males in the second reproductive cycle (RC) were fed a diet containing 5 or 2.5 μg EE_2_/g food for 28 days and then a commercial diet without EE_2_ for the remaining RC. Individuals were sampled at the end of the EE_2_ treatment and then at the end of the RC and at the beginning of the third RC, 146 and 333 days after the cessation of treatment, respectively. Increased hepatic transcript levels of the gene coding for vitellogenin (*vtg*) and plasma levels of Vtg indicated both concentrations of EE_2_ caused endocrine disruption. Modifications in the histological organization of the testis, germ cell proliferation, plasma levels of the sex steroids and pituitary expression levels of the genes coding for the gonadotropin β-subunits, *fshβ* and *lhβ* were detected. The plasma levels of Vtg and most of the reproductive parameters were restored 146 days after treatments. However, although 50% of the control fish underwent sex reversal as expected at the third RC, male-to female sex change was prevented by both EE_2_ concentrations.

## Introduction

Nowadays there is a broad variety of chemicals discharged from industrial and urban sources that have been described as endocrine disrupting chemicals (EDCs). EDCs exert adverse effects by means of their interactions with hormone receptors or by interfering with the normal synthesis, metabolism, transport, or secretion of endogenous hormones in wildlife and humans^1^. Among EDCs, 17α-ethynylestradiol (EE_2_) has received much attention in recent decades. EE_2_ is a synthetic estrogen that mimics E_2_ actions and is frequently used in contraceptive pills and hormone therapy and is widespread in aquatic environments^2–5^. Exposure to sub-lethal concentrations of EE_2_ causes alterations in reproductive capacity^6–14^ and sex differentiation^15–17^ in fish. The expression of the gene coding for vitellogenin (Vtg, a protein normally synthesized by females during oocyte maturation) and/or plasma levels of Vtg in male fish are reference markers widely used for estrogenic disruption^6–8,12^. Alterations in reproductive endpoints such as plasma levels of steroid hormones^7–9,12,13^, gonad morphology^6–9,13,14^ and the expression levels of genes coding for estrogenic receptors or for steroidogenic enzymes^8–10,13^ help to assess the disruptive effects of this compound which vary according to dose, time of exposure, age and the stage in the reproductive cycle of the treated individuals^7,8,17^. However, only a few studies have dealt with the effect of EE_2_ on the expression of genes coding for the β subunit of gonadotropins, luteinizing hormone (*lhb*) and follicle stimulating hormone (*fshb*)^13,18,19^, that are regulated by E_2_ in some species^20,21^ and are essential for regulation of gametogenesis and gonadal differentiation in fish.

Less attention has been paid to the potential for recovery from EE_2_ exposure and most studies are limited to assessment of sexual differentiation and the reproductive capacity of developing zebrafish, a fish model widely used in research. Reversal of the effects of EE_2_ exposure differed with the endpoint studied, the length of the exposure and the duration of the recovery period^17,22–24^. In addition, EE_2_ exposure has been reported to affect sex ratios^25^, to have transgenerational effects on fish survival and fecundity and to disrupt population dynamics of small model fish^17,26^.

The gilthead seabream (*Sparus aurata* L.) is a protandrous hermaphroditic fish species that develops as a functional male for the first 2 years of life and then 40% of a given population develops as females during the third year^27^. Little attention has been paid to the natural sex change in this species^27,28^ despite its great commercial and economic interest in the Mediterranean area^29^. Although the natural sex change in hermaphrodite fish has been the subject of numerous studies (review in^30–32^) most dealing with the male-to-female change have been carried out in the protandrous black porgy^20,33,34^.

Based on the status of the testis, the second reproductive cycle (RC) of the gilthead seabream can be divided into four main stages: spermatogenesis, spawning, post-spawning and testicular involution. During spermatogenesis, the testicular tubules contain spermatogonia stem cells and cysts of spermatogonia, spermatocytes and spermatids and varying amounts of spermatozoa exist in the lumen. At this stage, circulating levels of testosterone (T) and 11-ketotestosterone (11-KT) are high and 17β-estradiol (E_2_) is low. At spawning, the tubules of the testis contain mainly spermatogonia stem cells and cysts of primary spermatogonia and spermatozoa fill the lumen; circulating levels of T and 11-KT fall and E_2_ levels rise. At post-spawning, the tubules maintain the cell arrangement, but the lumen contains no, or very few, spermatozoa and serum E_2_ levels fall. At the testicular involution stage, tubules contain mainly spermatogonia stem cells and have no lumen and numerous necrotic areas surrounded by abundant interstitial tissue rich in eosinophilic cells appear in the internal testicular area, while serum levels of steroids remain low and constant^27,29^.

Previously we demonstrated that the effect of EE_2_-feeding on reproductive parameters of mature gilthead seabream varied with the concentration of EE_2_ and the stage at which it was applied during the RC. The lowest concentration tested, 5 µg EE_2_/g food^7,8,35^, is far higher than those routinely recorded in aquatic environments, which are usually below 50 ng/L^36^, but is similar to the highest concentration reported by Bicchi et al.^37^ in Italian surface waters collected downstream of a wastewater treatment plant (4381 ng/L). Furthermore, when EE_2_ exposure was followed by a recovery period of 25 days when fish were fed a standard diet, reproductive parameters did not recover^35^.

The aim of the present study was to determine whether in the protandrous gilthead seabream: (1) exposure to lower concentrations of EE_2_ than previously used (2.5 µg EE_2_/g food) affected reproductive parameters; (2) if longer periods of recovery after cessation of EE_2_ treatment (146 and 333 days) restored normal reproductive parameters; and (3) if exposure to EE_2_ affected the sex change in this hermaphrodite fish. For this reason, mature gilthead seabream males in spermatogenesis during the second RC were fed a commercial diet containing 2.5 or 5 µg EE_2_/g food for 28 days and then fed commercial diet without EE_2_ until the following RC. Fish were sampled after recovery for 146-days on a standard commercial diet, during the testicular involution stage when hormone levels are not affected by gametogenesis or spawning. Further samples were collected after 333 days of feeding on the standard commercial diet, at the beginning of the third RC when the sex change can be detected.

## Results

### EE_2_ did not affect body weight but disrupted the transcript levels of the *vtg* gene and the plasma levels of Vtg

**Figure 1:**
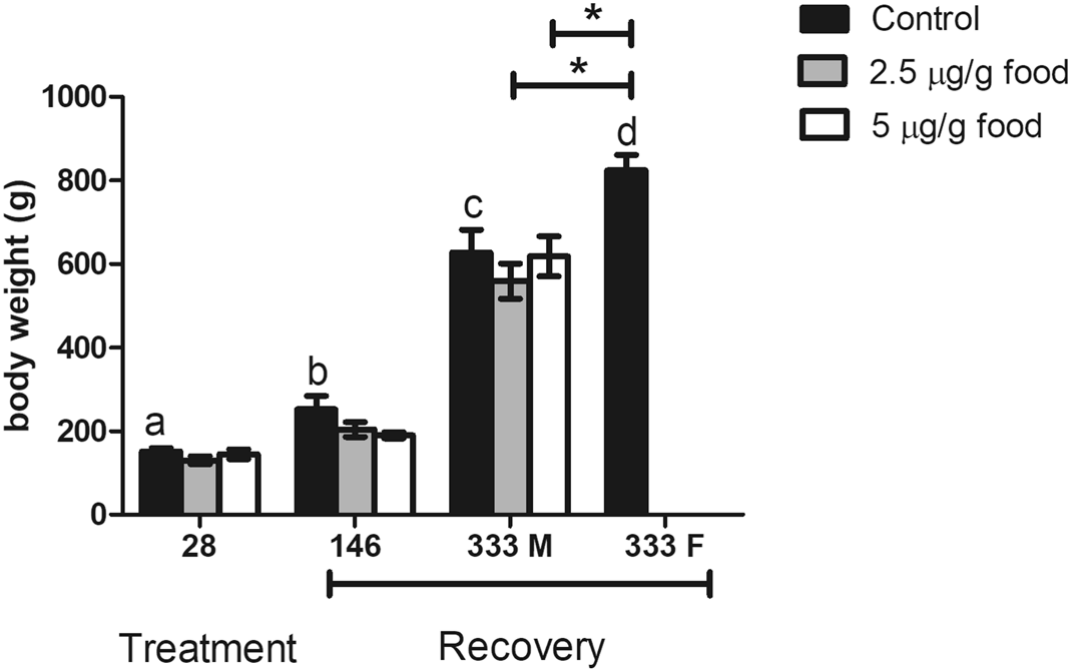
Effects of EE_2_ dietary intake on fish body weight after 28 days of treatment with 0 (control), 2.5 and 5 µg/g food, and after 28 days of treatment with 0 (control), 2.5 and 5 µg/g food followed by 146 and 333 days of feeding a standard diet (recovery). Data represent the means ± S.E.M. of duplicate samples. M, male specimens; F, female specimens. *Denote statistically significant differences between treated and control groups and letters indicate differences in the control group at distinct sampling times, as determined by one-way ANOVA and LSD post hoc tests.

Exposure to 2.5 or 5 μg EE_2_/g food for 28 days did not affect the fish body weight at the end of treatment or after 146 days of recovery using the standard diet. After 333 days recovery there were no significant differences between the weight of male control fish and the males that had been fed either 2.5 or 5 μg EE_2_/g food. The female control fish were significantly heavier than the control or EE_2_-treated males (Fig. 1).

**Figure 2:**
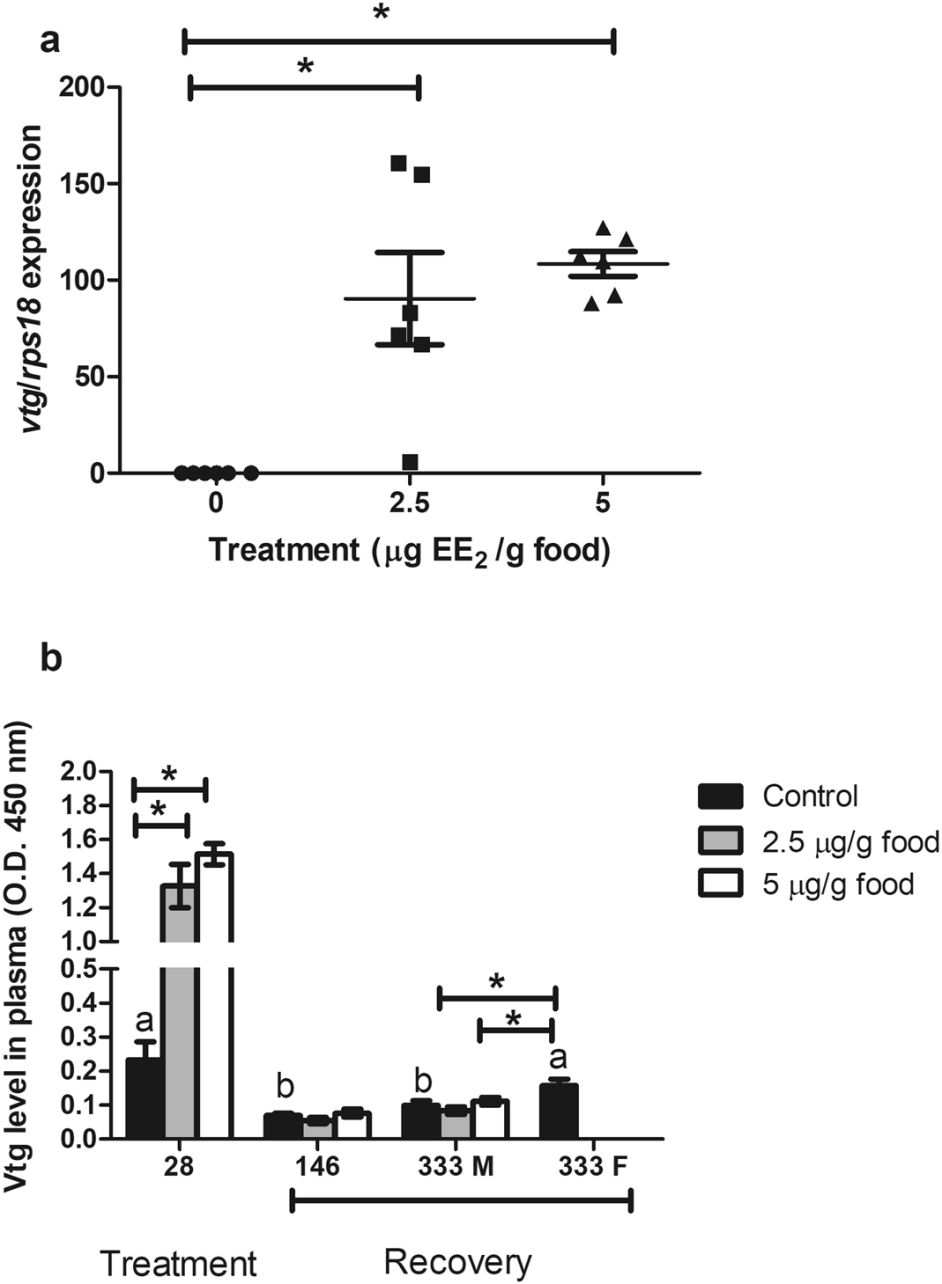
(**a**) Transcript levels of *vtg* gene in fish fed with EE_2_ at concentrations of 0 (control), 2.5 and 5 µg/g food for 28 days. (**b**) Plasma Vtg levels of fish fed 0 (control), 2.5 and 5 µg/g food for 28 days (treatment) and of fish fed 0 (control), 2.5 and 5 µg/g food for 28 days and afterwards a standard diet for 146 and 333 days (recovery). M, male specimens; F, female specimens. *Denote statistically significant differences between treated and control groups and letters indicate differences in the control group at distinct sampling times, as determined by one-way ANOVA and LSD post hoc tests.

Exposure to 2.5 or 5 μg EE_2_/g food for 28 days had disruptive effects, since it caused a significant increase in the hepatic expression levels of *vtg* (Fig. 2a) and plasma levels of Vtg compared to the control fish (Fig. 2b). The Vtg plasma levels were restored to control levels in all treated fish after a recovery period of 146 days (Fig. 2b). Interestingly, at day 333 when the sex change was accomplished in 50% of the control fish, the control females had increased plasma Vtg compared to the control and EE_2_ treated fish that maintained the male fate (Fig. 2b).

### The effect of a 28-days exposure to EE_2_ on the microscopic organization of the gonads varied according to concentration and specimens

**Figure 3:**
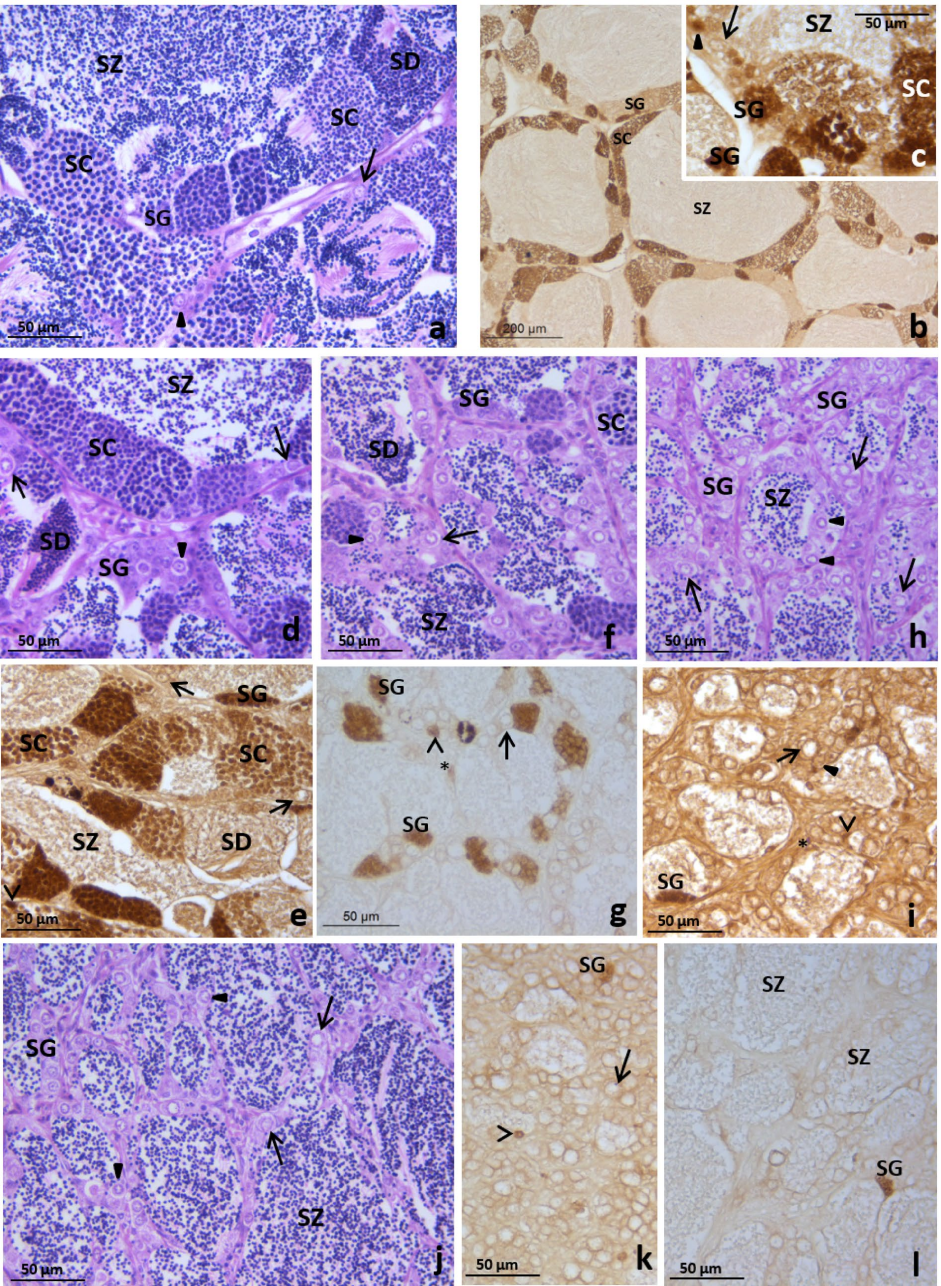
Testis of gilthead seabream fed with 0 (control, **a**–**c**), 2.5 (**d**–**i**) or 5 (**j**–**l**) µg of EE_2_/g of food for 28 days showing tubules with spermatogonia stem cells (arrow), primary spermatogonia (solid arrowhead) and cysts of spermatogonia (SG), spermatocytes (SC) and spermatids (SD with plenty of spermatozoa (SZ) in the lumen. Note that besides some interstitial cells (asterisk) and Sertoli cells (open arrowhead), diminished (**f**,**g**) or absent (**h**,**i**) cysts of proliferating germ cells were found in some individuals fed with 2.5 (**g**,**i**) and in those fed 5 µg of EE_2_/g of food (**j**–**l**). H–E (**a**,**d**,**f**,**h**,**j**); PCNA-immunolabelling (**b**,**c**,**e**,**g**,**i**,**k**,**l**).

At the end of a 28-day period of EE_2_-feeding, all the specimens in the control group displayed the morphological features (Fig. 3a) and cell proliferation pattern as revealed by anti-PCNA immunolabelling (Fig. 3b,c) typical of the late spermatogenesis stage in two-year old specimens^27^. In the testis tubules a germinal epithelium consisting of spermatogonia stem cells, primary spermatogonia (some of them proliferating), numerous cysts of proliferative spermatogonia and spermatocytes and non-proliferative spermatids were evident and abundant spermatozoa filled the lumen (Fig. 3). Fish fed with 2.5 μg EE_2_/g food showed variable levels of gonad tissue disruption (Fig. 3d–i). Thus, among the six specimens studied, half of them had a testicular morphology and proliferative pattern similar to those of control fish (Fig. 3d–e). However, one specimen displayed a decreased number of cysts and the anti-PCNA immunolabelling revealed a reduced number of proliferative spermatogonia and spermatocytes (Fig. 3f–g) compared to the control fish. The remaining two specimens fed 2.5 μg EE_2_/g food had testis resembling those of fish fed with a diet containing 5 μg EE_2_/g food (Fig. 3h–i) that are described below. The fish fed with 5 μg EE_2_/g food had a germinal epithelium lacking meiotic cells, with scarce proliferative spermatogonia, Sertoli and interstitial cells, and abundant spermatozoa filling the lumen of the tubules (Fig. 3j–l).

### No morphological alterations were observed in the testis of EE_2_-fed specimens after 146 days recovery on a standard diet

**Figure 4:**
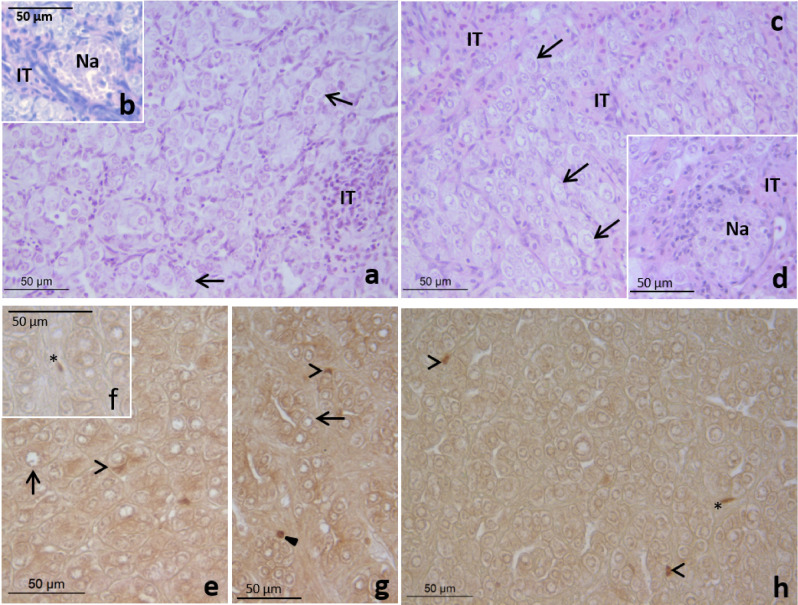
Testis of gilthead seabream fed with 0 (control, **a**,**b**,**e**,**f**), 2.5 (**d**,**g**) or 5 (**c**,**h**) µg of EE_2_/g of food for 28 days followed by a recovery period of 146 days of feeding with a standard diet showing collapsed tubules consisting of mainly spermatogonia stem cells (arrow) and primary spermatogonia (solid arrowhead), expanded interstitial tissue (IT) and necrotic areas (Na). Note the scarce proliferative interstitial (asterisk, **f**,**h**), Sertoli (open arrowhead, **e**,**h**) and germ (solid arrowhead, **g**) cells. H–E (**a**–**d**); PCNA-immunolabelling (**e**–**h**).

**Figure 5:**
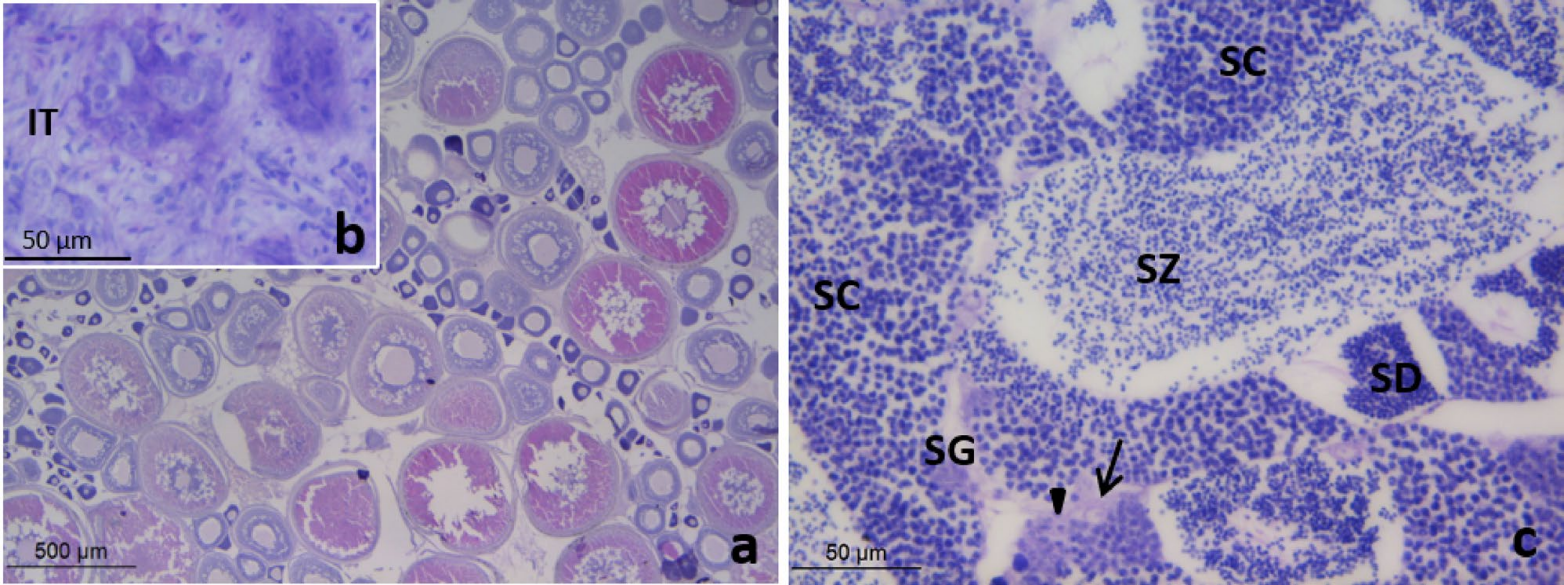
Gonads of gilthead seabream fed with 0 (control, **a**,**b**) and 2.5 µg of EE_2_/g of food for 28 days followed by a recovery period of 333 days of feeding with a standard diet (**c**). The control fish is a female and has an ovary with secondary oocytes (**a**) and shrunk testis with collapsed tubules consisting of dark cells and sparse spermatogonia stem cells (**b**). The treated fish showed testis at spermatogenesis (**c**) with tubules consisting of with spermatogonia stem cells (arrow), primary spermatogonia (solid arrowhead) and cysts of spermatogonia (SG), spermatocytes (SC) and spermatids (SD) and plenty of spermatozoa (SZ) in the lumen. H–E.

After 28 days of treatment and 146 days of recovery the testis of fish fed 2.5 (Fig. 4d) or 5 μg (Fig. 4c) EE_2_/g food had a similar morphology to the control fish with a histological organization typical of the testicular involution stage^27^ (Fig. 4a,b). The tubule lumen was very narrow or lacking and the germinal epithelium consisted mainly of spermatogonia stem cells, primary spermatogonia and rare cysts of spermatogonia and conspicuous necrotic areas were found in the inner testicular area (Fig. 4a–d). PCNA-immunolabelling revealed low proliferation of interstitial (Fig. 4f,h), Sertoli cells (Fig. 4e,g,h) and spermatogonia (Fig. 4g) in the control (Fig. 4e,f) and EE_2_ treated fish after 146 days recovery (Fig. 4g,h).

### Exposure to EE_2_ for 28 days during the spermatogenesis of the second RC prevented the sex change

At 333 days after the cessation of EE_2_ treatments half of the control fish had testis at the spermatogenesis stage (supplementary data). In the other 50% of the control fish the testicular tissue was a minor part of the gonad and contained collapsed tubules, consisting of dark cells and sparse spermatogonia stem cells; the ovarian area was dominant and contained abundant vitellogenic oocytes (Fig. 5a,b). However, the gonads of all the specimens that had been fed either 2.5 (Fig. 5c) or 5 μg (supplementary data) EE_2_/g food were similar to those of the control males at this stage.

### Sex steroid plasma levels were variable in EE_2_-fed fish and in recovered fish fed standard diets for 146 days

**Figure 6:**
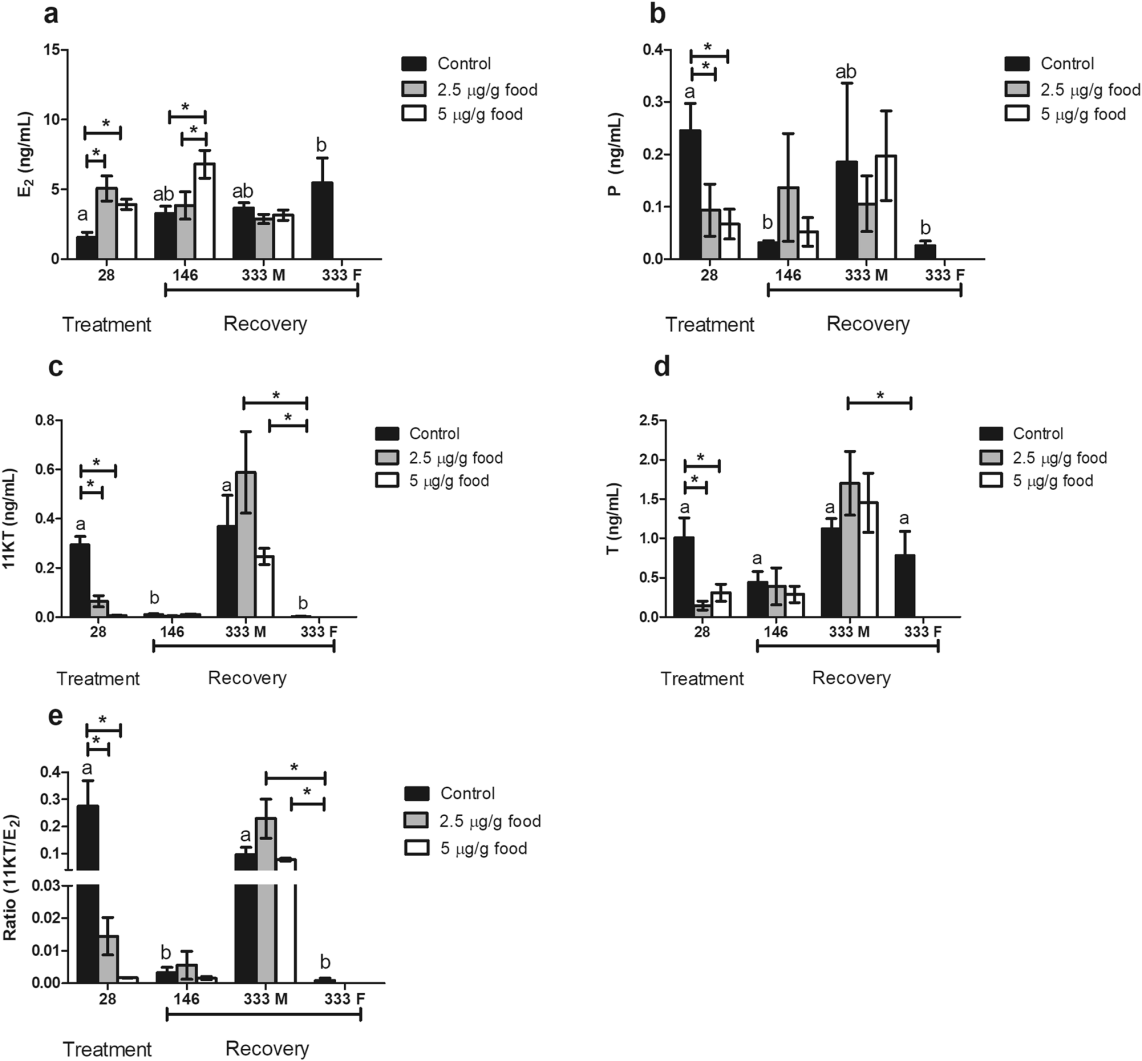
Plasma levels of 17β-estradiol (E_2_) (**a**), progesterone (P) (**b**), 11-ketotestosterone (11-KT) (**c**) and testosterone (T) (**d**) of fish fed 0 (control), 2.5 and 5 µg/g food for 28 days (treatment) and of fish fed 0 (control), 2.5 and 5 µg/g food for 28 days and afterwards a standard diet for 146 and 333 days (recovery). Data represent the means ± S.E.M. of duplicate samples. M, male specimens; F, female specimens. *Denote statistically significant differences between treated and control groups and letters indicate differences in the control group at distinct sampling times, as determined by one-way ANOVA and LSD post hoc tests (**a**–**d**) or Kruskal Wallis followed by Mann Whitney tests (**e**).

**Figure 7:**
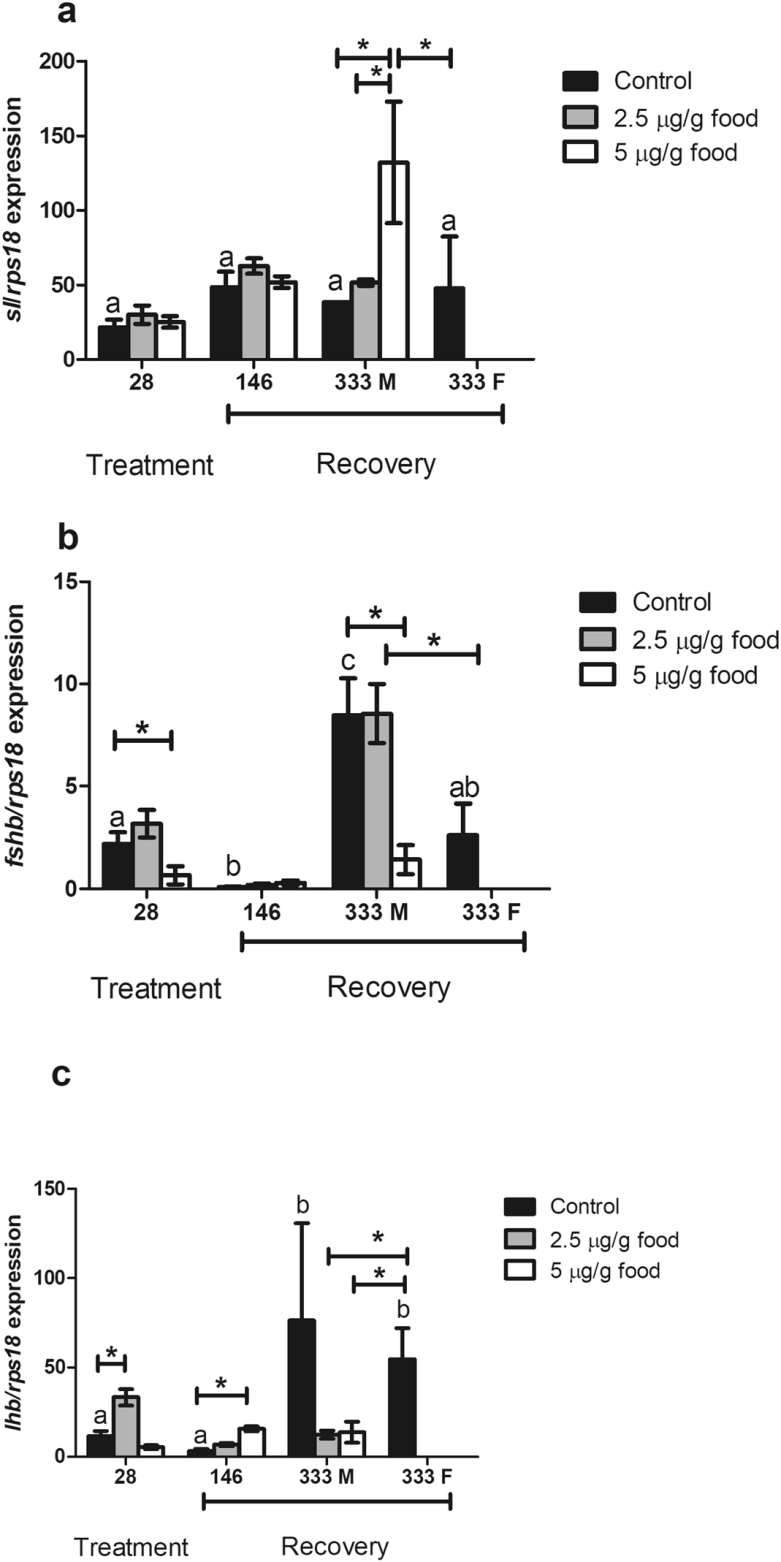
Transcript levels of *sl* (**a**), *fshb* (**b**) and *lhb* (**c**) gene in fish fed with EE_2_ at concentrations of 0 (control), 2.5 and 5 µg/g food during 28 days (treatment) and of fish fed 0 (control), 2.5 and 5 µg/g food for 28 days and afterwards a standard diet for 146 and 333 days (recovery). M, male specimens; F, female specimens. *Denote statistically significant differences between treated and control groups and letters indicate differences in the control group at distinct sampling times, as determined by one-way ANOVA and LSD post hoc tests.

Feeding with EE_2_ at concentrations of either 2.5 or 5 μg EE_2_/g food caused a significant increase in plasma levels of E_2_ (Fig. 6a) and a significant decrease in plasma levels of P (Fig. 6b), 11-KT (Fig. 7c) and T (Fig. 6d) leading to a ratio of 11-KT/E_2_ lower than the control fish (Fig. 6e). Control levels of sex steroids in EE_2_ treated fish were restored after 146 days of feeding a standard diet (Fig. 6b,d), with the exception of E_2_ which remained significantly elevated in fish that had been fed with 5 μg EE_2_/g food but not in fish fed with 2.5 μg EE_2_/g food (Fig. 6a). After 333 days recovery on a standard diet the sex steroid plasma levels of male EE_2_-fed fish did not differ from the male control. However, the control fish that developed as females had significantly lower levels of 11-KT and a lower 11-KT/E_2_ ratio than both control and treated males (Fig. 6a,e). Fish fed 2.5 μg EE_2_/g food had increased plasma T levels compared to control females.

### Gonadotropin gene transcripts were altered after 28 days of EE_2_ treatment and during recovery

The pituitary expression levels of *sl* were not affected by EE_2_ treatment at any dose (Fig. 7a). However, they were increased 333 days after cessation of treatment in fish fed 2.5 μg EE_2_/g food (Fig. 7a). Pituitary expression levels of *fshb* were significantly down-regulated after 28 days of 5 μg EE_2_/g food-intake and restored after 146 days of feeding with a standard diet. However, in males 333 days after cessation of feeding the 5 μg EE_2_/g food there was a significant down-regulation of *fshb* transcript abundance and levels resembled that of control females (Fig. 7b). Compared to the control fish the pituitary expression level of *lhb* was significantly up-regulated in fish fed 2.5 μg EE_2_/g food for 28 days (Fig. 7c) and also in fish at 146 days after cessation of feeding 5 μg EE_2_/g food (Fig. 7c). After a 333 days recovery period, *lhb* expression levels in EE_2_-fed fish were significantly lower than in control females (Fig. 7c) and were similar to those in control males.

### Sperm quality was similar in all males 333 days after the cessation of EE_2_ treatment

The concentration and viability of sperm from fish fed standard diets for 333 days after exposure to 2.5 or 5 μg EE_2_/g food during the spermatogenesis stage of the second RC were similar to control males at the spermatogenesis of the third RC (Table 1).

**Table1:** Effect of the dietary intake of 2.5 or 5 µg of EE_2_ g^−1^ of food for 28 days followed by 333 days recovery with a standard diet on the concentration (× 10^9^ cell/mL) and viability of sperm.

Treatment	Sperm concentration (× 10^9^ cell/mL)	Viability (%)
Control	6.21 ± 1.50	96.32 ± 0.73
2.5 µg of EE_2_ g^−1^ of food	6.77 ± 0.84	97.40 ± 0.30
5 µg of EE_2_ g^−1^ of food	6.73 ± 0.59	97.67 ± 0.31

Data represent the means ± S.E.M. of 6 males from each experimental group. No significant differences were identified (ANOVA test).

## Discussion

Exposure to 5 µg EE_2_/g food for 25 or 28 days had a disruptive effect in male gilthead seabream, since it increased the hepatic expression of *vtg* and disrupted spermatogenesis and steroidogenesis. The observed effects varied with the EE_2_ concentration, time of exposure, age and reproductive stage of the individuals^7,8,35^. Moreover, a 25-day recovery period with a standard diet after EE_2_ treatment did not restore the altered reproductive parameters in mature gilthead seabream^35^.

In the present study, mature gilthead seabream males at spermatogenesis of the second RC were used to investigate the disruptive effect of EE_2_ using a similar dose to our previous study and a lower dose (2.5 µg EE_2_/g food in diet). The effect was assessed by analyzing some parameters previously studied (histological organization of the testis, the plasma levels of the sex steroids E_2_, T and 11-KT and the sperm quality) and new selected reproductive endpoints, such as the plasma levels of P and the pituitary expression levels of the gene coding for SL, *sl*, a hormone related to reproductive activity^38–41^ and to sex differentiation^42^, and of the genes coding for the β-subunits of the gonadotropic hormones*, lhb* and *fshb*. Expression levels of *vtg* and/or plasma levels of Vtg were determined to assess the disruptive effects of EE_2_ treatments. Furthermore, the recovery for 146 and 333 days after EE_2_ exposure was investigated to explore whether the treatment caused disruption of the natural sex change of this species. Fish that recovered from EE_2_ treatment for 146 days were at the end of the RC, when the brain-pituitary-gonad axis is not conditioned by the course of gametogenesis or spawning and many fishes change sex^30^. Fish that recovered from EE_2_ treatment for 333 days were undergoing the gametogenesis of the third RC, when approximately 40% of the gilthead seabream normally develop as females^27^, which was confirmed in the present study, for the control fish only.

Both doses of EE_2_ had a disruptive effect on gilthead seabream reproduction as revealed by increased hepatic *vtg* gene expression and plasma Vtg and increased plasma E_2_ and decreased plasma T, 11-KT and P compared to the control. Modifications in plasma E_2_, T and 11-KT in response to EE_2_ exposure in fish^12,43,44^ including the gilthead seabream, depends on the reproductive stage at which it occurs^7,8^. The effect of EE_2_ on the testis histology depended on the dose and higher doses had a more pronounced effect in common with previous reports in the gilthead seabream^8,35^ and other teleost fish^13,45^. Although in our previous study we reported that 5 µg EE_2_/g food caused a reduction in the sperm concentration in gilthead seabream at the spermatogenesis stage^35^. It was not possible to obtain sperm samples from the fish fed 5 µg EE_2_/g food in the present study, although similar observations have been made in pejerrey mature males exposed to 45 ng EE_2_/L during the spawning season^13^.

Less attention has been paid to the effect of EE_2_ exposure on pituitary hormones^13,18,19^. The doses used in this study had a variable effect on pituitary expression levels of *fshb* (decreased with 5 µg EE_2_/g food) and *lhb* (increased by 2.5 µg EE_2_/g food). Similar observations have also been made in sub-adult female coho salmon (*Oncorhynchus kisutch*) with EE_2_ exposure (12 ng EE_2_/L for 6 weeks) causing a high increase in the expression of *lhb* and a moderate decrease in the expression of *fshb*^18^. In adult *Gobio cyprisrarus* of both sexes, mRNA levels of *fshb* were not affected and *lhb* gene transcripts were suppressed by 1–125 ng EE_2_/L^19^. In contrast, in pejerrey (*Odontesthes bonariensis*) exposure of mature males to 45 ng/L of EE_2_ did not affect pituitary expression levels of *lhb or fshb*^13^.

In gilthead seabream, most effects of EE_2_ on the reproduction-related endpoints studied were lost 146 days after the cessation of treatments, including the Vtg plasma levels used as marker of endocrine disruption. However, gilthead seabream were not fully recovered from EE_2_ exposure, as some alterations were observed at the pituitary level 146 and 333 days after the cessation of exposure to 5 µg EE_2_/g food. In addition, all fish had testis at spermatogenesis 333-days after cessation of EE_2_ exposure irrespective of the dose (2.5 or 5 µg EE_2_/g food), although in half of the control fish the histological changes were consistent with the sex change that naturally occurrs in this species^27^. Thus, the exposure to EE_2_ of adult gilthead seabream for a short period during the second RC produced long-lasting disruptive effects. Studies on zebrafish exposed to EE_2_ from fertilization to sexual maturation described partial reversal of the feminizing effects^24^ and the reproductive consequences^22^ eight and five months after cessation of exposure. In the same species a 3-month depuration following EE_2_ exposure throughout development was not enough to restore control levels of Vtg synthesis and gonad morphololy^23^, although a total rescue from underdevelopment and feminization was described 40 days after the cessation of EE_2_ exposure under similar conditions^17^.

On the other hand, the effects of EDCs in the sex ratio of gonochoric laboratory fish models may be transitory or permanent and timing for determining fish sex after exposure is important^25^. Hence, studies on a variety of species and conditions are needed to fully understand the reversibility of the endocrine disruptive effects of EE_2_. Research on hermaphroditic marine fish as the gilthead seabream would be very valuable, especially considering its great economic interest in the Mediterranean area.

Noticeably, sex change failed to occur in fish treated with 2.5 or 5 µg EE_2_/g food, whilst as expected 50% of control specimens became females^27^. The progression of natural sex change involves concomitant changes in E_2_ synthesis in many species, with levels increasing in protandrous^46,47^ and dropping in protogynous^48^ fish. In the protandrous sobaity (*Sparidentex hasta*) the attainment of a minimum threshold level of E_2_ that enables the ovarian portion of the gonad to establish temporary dominance and facilitate further establishment of a permanent female status seems to be necessary for sex change to occur^49^. On the other hand, it has been proposed a deficiency in gonadal production of E_2_ is the signal resulting in the failure of sex change in the protandrous black porgy^50^. In the present study, an increase in the plasma levels of E_2_ was not detected in control fish, which were similar at spermatogenesis and at testicular involution stages of the second RC. In addition, treatments with both doses of EE_2_ elevated plasma levels of E_2_ but only fish treated with 5 µg EE_2_/g food maintained these high levels after a 146-day recovery. However, all the EE_2_ treated fish irrespective of dose remained male in the following RC. Thus, the increase of E_2_ plasma levels does not seem to be essential to stimulate sex change in gilthead seabream. Protandrous transition involves, besides the elevation of E_2_ levels, the cease of 11-KT synthesis, and a subsequent proliferation of ovarian and degeneration of testis, respectively^30^. In gilthead seabream, a drop in plasma levels of 11-KT was observed in control fish at the testicular involution stage compared to control fish at spermatogenesis. However, although both EE_2_ treatments caused a drop in 11-KT plasma levels, which recovered at 146 days so fish had 11-KT plasma levels similar to the control fish at the testicular involution stage, sex change did not occur in any of the EE_2_-treated fish. Thus, the occurrence of low 11-KT plasma levels does not seem to be determinant for sex change in the gilthead seabream. On the other hand, our data corroborates the outcome of previous studies that have failed to directly link T and P to sex change. Hence, unlike other teleost fish^46–50^, steroid levels do not seem to be key triggers of sex change in the gilthead sea bream.

Gonadotropins seem to be involved in protandrous sex change, although their role is not well understood. In clownfish induced to change sex by removing females, expression levels of both *fshb* and *lhb* increased as the sex change progressed^51^. However, in the black porgy the role of gonadotropins is controversial. Lower expression levels of *fshb* and *lhb* are detected during natural sex change from male to females^20^. In contrast, increased expression levels of *lhb* have been described in the fish undergoing male-to-female and female-to-male induced sex change^52^. Furthermore, a rise in plasma levels of LH has been recorded in male fish compared to fish changing sex following the second spawning period and before recrudescence of the third RC in this species (June–July^50^, May–August^53^, April–June^54^). Likewise, in the present study the pituitary expression levels of *fshb* were lower in control gilthead seabream at the testicular involution stage than at spermatogenesis of both the second and the third RCs. However, EE_2_ affected this differential expression only in fish fed 5 µg EE_2_/g food. Since fish of both experimental groups maintained the male status for the following RC, the alterations of expression levels of *fshb* does not seem to be the cause of the failure of sex change in EE_2_-exposed gilthead seabream. On the other hand, on this study, control fish showed similarly low expression levels of *lhb* in pituitary at spermatogenesis and testicular involution stages of the second RC and similarly high pituitary expression levels of *lhb* in control males and females at the spermatogenesis of the third RC. As a result of EE_2_ exposure, pituitary expression levels of *lhb* were increased upon treatment in fish fed 2.5 µg EE_2_/g food and upon feeding and recovering for 146 days in fish fed 5 µg EE_2_/g food, compared to control fish. This increased LH signal could maintain the male fate and avoid the development of femaleness, as proposed for black porgy^55^. In this species, LH signal seems to preserve male fate^55^, throughout the maintenance of *dmrt1* expression^54,55^, whilst femaleness would be switched on by the regression of testicular development as a result of the diminished *dmrt1* expression levels in testis^54,55^. In gilthead seabream it has been reported that expression levels of *dmrt1* decrease during the testicular involution phase prior to sex change^27^. Moreover, exposition to 5 µg EE_2_/g food in diet during spermatogenesis resulted in a decrease in expression levels of *dmrt1* in the testis of mature gilthead seabream that persisted after 25 days of recovery with a standard diet^35^.

Hence, the exposure of gilthead seabream to EE_2_ in the diet (2.5 or 5 µg EE_2_/g food) for a short period of time (28 days) at the spermatogenesis stage of the second RC caused alterations that could be ascertained a long time after (146 days) in E_2_ plasma levels and even longer (333 days) in expression levels of genes coding for pituitary hormones involved in the regulation of reproductive events. However, these disturbances could not be directly correlated to the failure of sex change because they were dissimilar in fish fed the two EE_2_ doses employed, while both treatments prevented sex change.

Taking into account all these data, it could be suggested that in gilthead seabream there is a wide period of gonadal sensitivity in which exogenous factors, such as endocrine disruptors or sex steroids, could affect the natural sex change in a way similar to the critical period of gonadal differentiation during which exogenous sex hormones can induce the reversal of the determined sex in several fish species^56–58^. This agrees with the report in black porgy of a sensitive period for E_2_ treatment to achieve controlled sex change from early pre-spawning to the spawning season^50^. Thus, the period in which sex change is determined in gilthead seabream seems to be longer than just the testicular involution stage meaning that sex change could be impaired during an extensive period of the RC. However, the possibility of a delayed effect of treatments on some key factor in triggering the sex change in this species cannot be discarded. Thus, there is a long way to go to fully understand the mechanism behind the EE_2_ effect on sex change in gilthead seabream.

On the other hand, there is a close relationship between weight, sex and estrogens in fish having sex-linked growth dimorphism such as the tilapia^59^. In several protandrous fish species, including the gilthead seabream, sex change is attained at a bigger size than the achievement of sexual maturity in males (*Amphiprion sp.*^60^*, Lithognathus mormyrus*^61^, *Sparus aurata*^62^). In addition, in gilthead seabream quantitative trait loci involved in sex determination and in body growth have been detected in the same linkage group by genetic mapping, thus providing a genetic correlation between body weight and sex reversal in this species^63^. On the other hand, exposure to environmental estrogens has been associated with impairment of the GH/IGF system in juvenile rainbow trout, *Oncorhynchus mykiss*^64^, and altered GH and IGF1 expression in the brain of developing tilapia (*Oreochromis niloticus*), and a significant reduction in growth of males^65^. However, in the present study EE_2_ treatment did not affect the weight of gilthead sea bream during the treatment or after the recovery period of 146 days. Thus, although in the present study, a clear relationship between sex and weight is evident, differences in weight between male and female gilthead seabream at the beginning of the third RC could respond to physiological requirements more than to the existence of a threshold size for triggering sex change in this species.

In conclusion, disruption in reproductive endpoints caused by EE_2_ treatments during spermatogenesis of the second RC in gilthead seabream lasted long beyond the cessation of exposure to the disruptive factor and resulted in failure of the natural sex change normally occurring at the third RC^27^. These results suggest that short-term, occasional exposure to endocrine disrupting chemicals can induce persistent alterations in fish, which would not be detected using the usual indicators of disruption, and can compromise the reproductive success in fish. Thus, studies on the long-term effects of endocrine disruptors are necessary to fully understand the consequences for reproduction and sex reversal of exposure to these compounds.

## Material and methods

**Figure 8:**
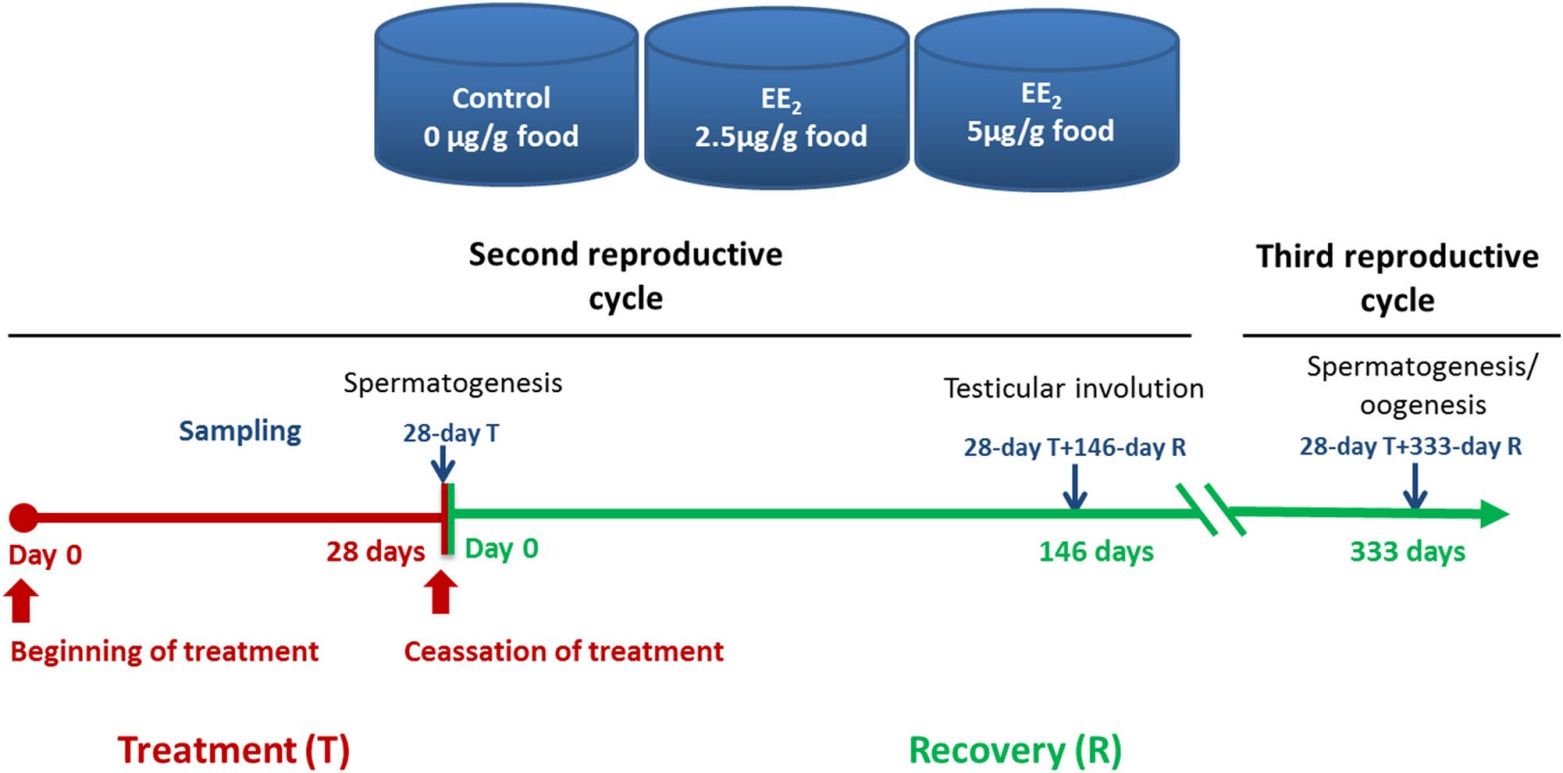
Schema of the experimental design. EE_2_, 17α-ethynylestradiol. Fish reproductive stages according to Liarte et al.^27^.

Male specimens of gilthead seabream (Actinopterygii, Perciformes, Sparidae) at the spermatogenesis stage of the second RC were kept under a natural photoperiod and water temperature ranging from 14.6 to 17.8 °C in 2 m^3^ tanks with a flow-through circuit and a suitable aeration and filtration system at the “Centro Oceanográfico de Murcia” (Instituto Español de Oceanografía, Spain). Specimens (n = 72) weighing 107–181 g body weight were fed a standard commercial diet (44% protein, 22% lipids, Skretting, Spain) containing 2.5 or 5 μg EE_2_/g food for 28 days. The EE_2_ (98% purity; Sigma) was incorporated using the ethanol evaporation method (0.3 L ethanol/kg of food) described by Shved et al.^66^. Fish were then fed the standard commercial diet without EE_2_ for 333 days. Fish were fed ad libitum three times a day and fasted for 24 h before sampling. Samples were collected at the end of the EE_2_ treatment (day 28) and at 146 and 333 days after cessation of EE_2_ treatment (n = 6 fish/group and time point, except at the last sampling when 6 additional male fish/group were used to obtain sperm). The control fish were fed the standard commercial diet without EE_2_ for the entire experiment (Fig. 8).

For sampling, specimens were anesthetized with 40 μL/L of clove oil in seawater and weighed, the urogenital pore dried before collection of sperm (described below). Trunk blood was extracted from the caudal peduncle using ammonium-heparinized syringes and fish were decapitated. The gonads were removed, weighed and processed for light microscopy, as described below. The pituitary and a fragment of liver were processed for gene analysis, as described below. Plasma samples were obtained by centrifugation of trunk blood, the red blood cells discarded and the plasma immediately frozen and stored at − 80 °C until use.

### Analysis of gene expression

Total RNA was extracted from the pituitary and the liver with TRIzol Reagent (Invitrogen, Barcelona, Spain) following the manufacturer’s instructions. Extracted total RNA was quantified with a spectrophotometer (NanoDrop, ND-1000) and the RNA of 6 fish/group was independently treated with DNase I (amplification grade, 1 unit/μg RNA, Invitrogen, Barcelona, Spain) to remove genomic DNA contamination. SuperScript III RNase H-Reverse Transcriptase (Invitrogen, Barcelona, Spain) was used to synthesize first strand cDNA from 1 μg of total RNA with an oligo-dT18 primer, at 50 °C for 50 min.

Real-time PCR performed with an ABI PRISM 7500 (Applied Biosystems, Madrid, Spain) using SYBR Green PCR Core Reagents (Applied Biosystems, Madrid, Spain). The expression of the genes coding for hepatic vitellogenin (Vtg, *vtg*), the pituitary hormone somatolactin (SL, *sl*) and the β-subunit of follicle stimulating hormone (FSH, *fshb*) and of luteinizing hormone (LH, *lhb*) were quantified. For each mRNA, gene expression was normalized to the ribosomal protein S18 gene (*rsp18*) was used to normalize samples and gene expression in each sample was determined using the comparative Ct method (2^−ΔΔCt^) (where Ct is a cycle threshold). The specific primers used in qPCRare shown in Table 2. Triplicate technical replicates were performed for all reactions.

**Table 2:** Gene accession numbers and the primer sequences used for gene expression analysis by real time PCR.

Gene	Accession number*	F/R	Sequence (5′–3′)
*vtg*	AF21042	F	CTGCTGAAGAGGGACCAGAC
		R	TTGCCTGCAGGATGATGATA
*sl*	L49205	F	AATAAATTCCTGGTAGCCGTCC
		R	CACTTTGCCCTCGTTGCCT
*fshb*	*	F	AGGACAGTGCTACCACGAGGAT
		R	CCGCAGTCCGTGTTTCCAG
*lhb*	*	F	AGAAGGAGGGCTGTCCCAAGT
		R	TGTAGTGTAAGTCCCGGTATGTGC

*Provided by Deborah Power.

### Analytical techniques

Plasma levels of Vtg were determined using an indirect enzyme-linked immunosorbent assay with a commercial rabbit polyclonal antibody against gilthead seabream Vtg (Ab, Biosense Laboratories). Progressive serial dilutions of plasma (1:50, 1:100, 1:500 and 1:1000) and anti-Vtg serum (1:200, 1:1000, 1:2000, 1:3000) were used to determine the linearity of serum dilution curve and the specificity of the antibody. The validation was performed twice and the intra- and inter-assay coefficients were calculated, obtaining 4.72 ± 1.06% and 3.91 ± 0.77%, respectively.

Plasma of control and EE_2_-treated fish (n = 6/ treatment/sampling time) at the optimal dilution of 1:500 in the coating buffer (sodium carbonate-sodium hydrogen carbonate, pH 9.6) were added to flat-bottomed 96-well plates for 24 h at 4 °C. Non-specific binding sites were blocked using 3% skimmed milk in phosphate-buffered saline (PBS) pH 7.3. Wells were incubated with the specific primary Ab at the optimal dilution of 1:1000 in PBS, for 1 h at room temperature and then with an anti-rabbit IgG conjugated with peroxidase diluted 1:1000 in PBS for 1 h at room temperature. Peroxidase activity was quantified by adding the chromogen solution (10 mM 3,3′,5,5′-tetramethylbenzidine (TMB) containing 0.015% H_2_O_2_). After 15 min of incubation, the reaction was stopped with 2 M H_2_SO_4_ and the absorbance was read at 450 nm using a FLUOstart luminometer (BGM, Lab Technologies). Data were determined by subtracting the absorbance of negative control wells, lacking Ab incubation, from the values obtained. The appraisals were performed in triplicate.

Plasma levels of T, 11-KT, E_2_ and progesterone (P) were quantified by ELISA following the method described by Rodríguez et al.^67^ and previously used in gilthead seabream^29^. Steroids were extracted from 20 μL of serum in 0.6 mL of methanol (Panreac). The methanol was then evaporated at 37 °C and the steroids were dissolved in 400 μL of reaction buffer [0.1 M phosphate buffer with 1 mM EDTA (Sigma), 0.4 M NaCl (Sigma), 1.5 mM NaN_3_ (Sigma) and 0.1% bovine serum albumin (Sigma)]. Then, 50 μL of each sample (1/8 dilution) was used for quantification in duplicates wells of the 96 multi-well ELISA plate (MaxiSorp, Nunc). The P, T, 11-KT and E_2_ standards, mouse anti-rabbit IgG monoclonal antibody (mAb), and specific anti-steroid antibodies and enzymatic tracers (steroid acetylcholinesterase conjugates) were obtained from Cayman Chemical. A standard curve for each of the steroids being quantified was prepared using serial dilutions from 6.13 × 10^−4^ to 2.5 ng/mL (0.03–125 pg/well) and included in each assay. Standards and extracted serum samples were run in duplicate. The lower limit of detection for all the assays was 12.21 pg/mL. The intra-assay coefficients of variation (calculated from duplicate samples) were 8.26% ± 1.33% for T, 8.80% ± 1.68% for 11-KT, 3.98% ± 0.57% for E_2_ and 7.92% ± 2.92% for P. The inter-assay coefficient of variation was determined at 50% of binding and was 14% for T (n = 2 assays), 4% for 11-KT (n = 3 assays), 34% for E_2_ (n = 4 assays) and 57% for P (n = 3 assays). Details about the cross-reactivity of each of the antibodies were provided by the supplier and for anti-T cross-reaction with 11-KT was 2.2%; for anti-11-KT cross-reaction with T was 0.01%; for anti-E_2_ reaction with T was 0.1% and for anti-P cross-reaction with E_2_ was 7.2%.

### Measurement of the sperm concentration and motility

Stripped sperm was collected from the genital pore with a syringe after applying gentle pressure on the abdomen so that the semen flowed out (urine-contaminated samples were discarded). The total sperm fluid from each fish (n = 6 fish/group and time) was used immediately to determine the sperm cell concentration and viability. As 333 days after the cessation of EE_2_ exposure, half of the control fish (n = 3) were found to be females, sperm was obtained from another set of 18 fishes (n = 6 males/group) after discarding females from the control group. To determine the sperm concentration, semen was diluted in 1% formol (Panreac) and 5% NaHCO_3_ (Sigma) in water at a ratio of 1:400 and the spermatozoa were counted using a Neubauer chamber. To determine the sperm viability, the propidium iodide staining method and a flow cytometer (BD Biosciences) were used.

### Light microscopy and immunocytochemical staining

The gonads were fixed in Bouin’s solution, embedded in Paraplast Plus (Sherwood Medical, Athy, Ireland), and sectioned at 5 µm using a Leizt rotary microtome. After dewaxing and rehydration, some sections were stained with hematoxylin–eosin in order to determine the reproductive stage of specimens. Some sections were used to analyze cell proliferation by immunocytochemistry using a commercial rabbit polyclonal antibody specific to proliferating cell nuclear antigen (PCNA, Sigma) that has previously been shown to cross-reacts with PCNA from all vertebrate species investigated so far, including fish^68^. No immunostaining was observed when the anti-PCNA sera was omitted from the immunocytochemical procedure.

### Calculation and statistics

The data related to *vtg* gene expression were analysed using a Student t-test to determine differences between the untreated control and the treated groups. The rest of the data were analysed using a one-way ANOVA and a post hoc test (Fisher Least significant difference, LSD) to determine differences between groups. The normality of the data was assessed using a Shapiro–Wilk test and homogeneity of variance was also verified using the Levene test. Non-normally distributed data were log-transformed prior to ANOVA analysis. In a few cases (the ratio 11-KT/E_2_) the transformed data did not meet the parametric assumptions and in these cases, the non-transformed data were analysed using a non-parametric Kruskal Wallis test, followed by a pair wise Mann Whitney test. The critical value for statistical significance was taken as *p* ≤ 0.05. Statistical analyses were conducted using Statgraphics 15.0 (StatPoint, Inc) and SPSS software. All data are presented as a mean of standard error to the mean (SEM).

### Ethical approval

Handling of specimens and methods used in this study were carried out in accordance with the Guidelines of the European Union Council (2010/63/UE).

All experimental protocols were approved by the Bioethical Committee of the IEO (reference REGA ES300261040017) and the “Consejería de Agua, Agricultura y Medio Ambiente” of the “Región de Murcia”, Spain (Approval Number A13160507).

## Supplementary information

Supplementary Figure 1.

## Data Availability

All data are available upon request, please contact Dr. Maria del Pilar García Hernández (e-mail: piligar@um.es).
