# Supplementary material for: 17α-ethynylestradiol prevents the natural male-to-female sex change in gilthead seabream (Sparus aurata L.)
Source: Sci Rep. 2020 Nov 18;10:20067. doi: 10.1038/s41598-020-76902-9 (PMC7676269; doi:10.1038/s41598-020-76902-9)
Supplement: Supplementary file 1 — Supplementary Figure 1. [file 41598_2020_76902_MOESM1_ESM.pdf]

17α-ethynylestradiol prevents the natural male-to-female sex change in gilthead seabream (*Sparus aurata* L.)

M. Pilar García Hernández[1]*, Isabel Cabas[1], M. Carmen Rodenas[1], Marta Arizcun[2] Elena Chaves-Pozo[2], Deborah M. Power[3], Alfonsa García Ayala[1]

Supplementary data:

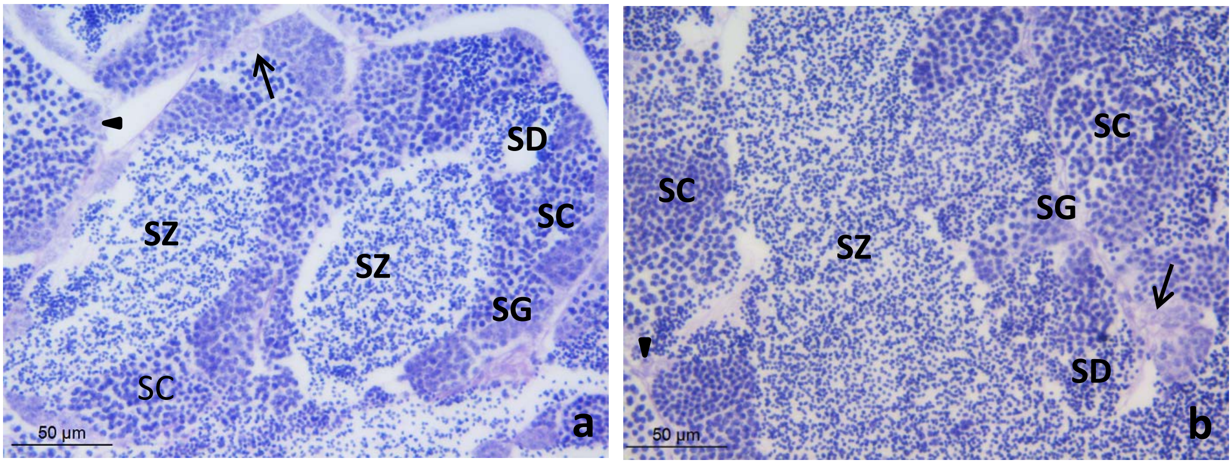

a-b. Gonads of male gilthead seabream fed with 0 (control, **a**) or 5 (**b**) µg of EE_2/g of food for 28 days followed by a recovery period of 333 days of feeding with a standard diet. Both specimen show testis at the spermatogenesis stage with tubules consisting of spermatogonia stem cells (↑), primary spermatogonia (▼) and cysts of spermatogonia (SG), spermatocytes (SC) and spermatids (SD and plenty of spermatozoa (SZ) in the lumen. H-E.
